# Assembloid learning: opportunities and challenges for personalized approaches to brain functioning in health and disease

**DOI:** 10.3389/frai.2024.1385871

**Published:** 2024-04-19

**Authors:** Arianna Mencattini, Elena Daprati, David Della-Morte, Fiorella Guadagni, Federica Sangiuolo, Eugenio Martinelli

**Affiliations:** ^1^Department of Electronic Engineering, University of Rome Tor Vergata, Rome, Italy; ^2^Interdisciplinary Center of Advanced Study of Organ-on-Chip and Lab-on-Chip Applications (IC-LOC), University of Rome Tor Vergata, Rome, Italy; ^3^Department of System Medicine and Centro di Biomedicina Spaziale (CBMS), University of Rome Tor Vergata, Rome, Italy; ^4^San Raffaele Rome University, Rome, Italy; ^5^Istituto di Ricovero e Cura a Carattere Scientifico (IRCCS) San Raffaele, Rome, Italy; ^6^Department of Biomedicine and Prevention, University of Rome Tor Vergata, Rome, Italy

**Keywords:** organoid intelligence, assembloid learning, brain organoids (BOs), machine learning (ML), personalized medicine, neurodegenerative diseases

## Introduction

One of the most remarkable properties of the brain is the ability to construct representations of the external environment, which can be used to simulate and plan future interactions. In the past 50 years, neuroscientists have devised novel techniques to observe, understand and modulate this capacity, either by visualizing neural activity *in vivo* or by recording and stimulating the brain via electrodes or electromagnetic fields. This effort has been invaluable also in advancing research on artificial intelligence (AI) and a strong crosstalk has ensued from which both domains have already benefited. Progresses in neural network design have laid the basis for using AI to identify anomalies in brain functioning and modeling neurological disorders but successful computer-assisted therapy as well as a complete understanding of how these diseases arise and evolve are far from acquired (Macpherson et al., 2021). Yet, based on 2016 data, these disorders are the leading cause of disability and second leading cause of death. In the US, approximately one in six children is born with a neurodevelopmental disorder (Mencattini et al., 2018), and 6.5 million individuals aged 65 and older suffer from Alzheimer's disease, a number only destined to increase (Eichmueller, 2022). Hence, to tackle these silent pandemics, we need new, “outside-the-box” research tools and strategies that allow us to devise highly personalized approaches (Kanai and Rees, 2011). In this paper, we first present the possible advantages offered by brain organoids (i.e., engineered cell-based *in vitro* models of *in vivo* tissues) and assembloids (i.e., 3D, self-organizing structures that functionally combine two or more organoids, allowing to model interactions between different tissues or regions) as additional tools to track and model neural activity, particularly with reference to complex functions like learning and memory. Next, we approach the methodological problems that still need to be solved before these novel instruments can be successfully implemented, examining the possible limits of an organoid-based approach to neurocognitive research. Finally, we discuss the ethical issues raised by the peculiar nature of brain organoids, briefly summarizing some proactive interventions that should be considered when engaging in this type of experimentation.

### Organoids-based models of brain function: what they can do and what they may learn to do

**Figure 1:**
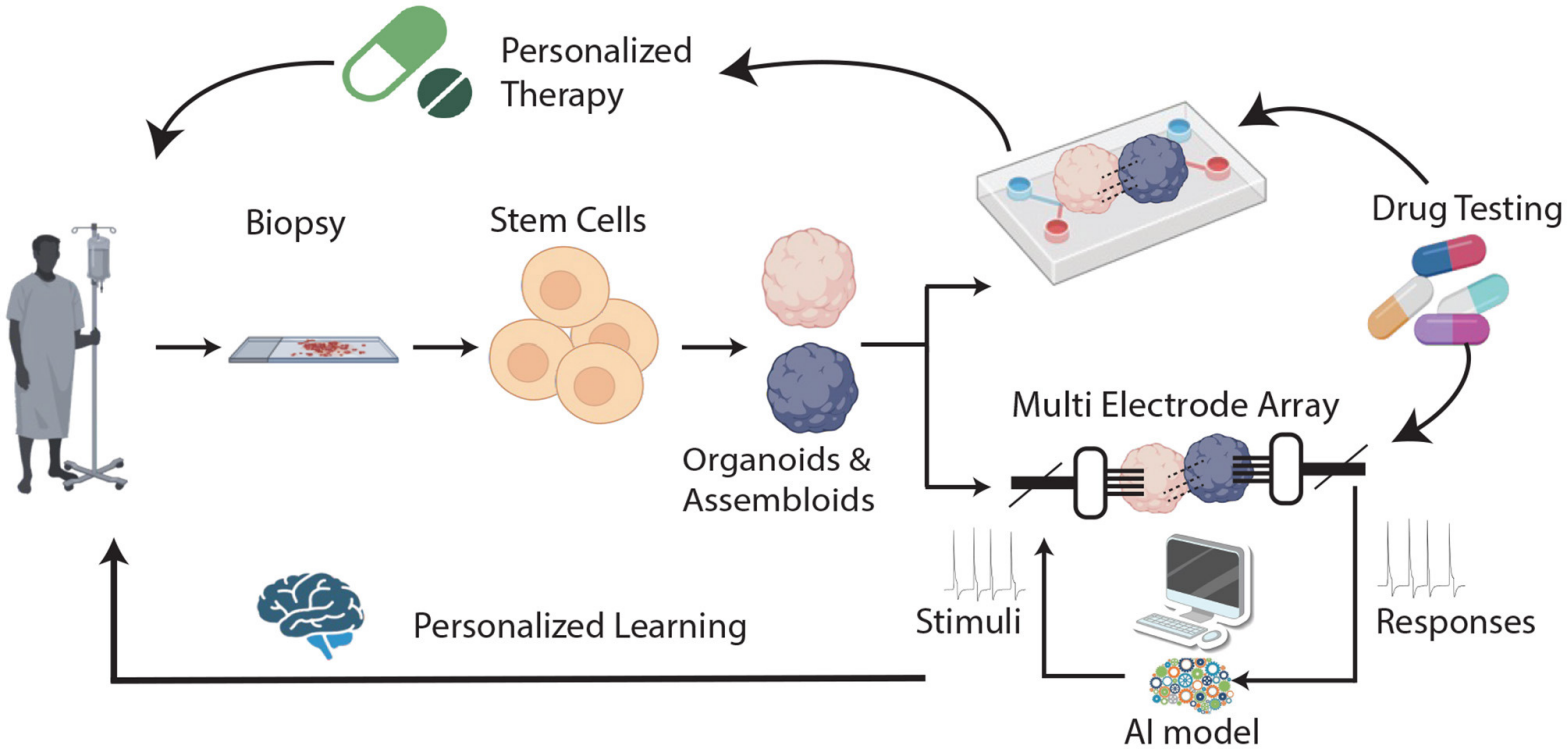
A general scheme of the proposed scenario. In the standard view, BOAs are cultured in an organ-on-chip (OOC) device where drug testing for personalized therapy can be performed **(upper branch)**. In the novel scenario, BOAs are also interfaced with a MEA **(lower branch)**: input and output electrical stimuli are manipulated through artificial intelligence (AI model) and used in learning and memory BOA tasks, leading to the possibility of developing personalized models of learning.

The rapid development of organoids and assembloids—cultures capable of arranging stem cells into specific 3D structures (Zhong et al., 2014; Bartfeld and Clevers, 2015; Bartfeld et al., 2015; Morizane et al., 2015; Wallach and Bayrer, 2017; Pleguezuelos-Manzano et al., 2020; Lawlor et al., 2021)—underscores the need to evaluate their strengths and limitations as innovative methods for studying body functions, developing treatments, and creating new drugs. In contrast to the traditional 2D tissue cultures (D'Orazio et al., 2022), organoids possess an inherent complexity, replicate the intricate cellular compositions and 3D architectures of natural organs (Hoang and Ma, 2021; Shi et al., 2024) and mimic the genetic makeup and physiological characteristics of individual patients. Brain organoids/assembloids (BOA), which originate from the growing, dynamic collaboration between stem-cell biology and bioengineering, are not organs and may not entirely replicate the organ of interest (Jensen and Little, 2023), but they can successfully recapitulate some organ-specific functions. Accordingly, they are possibly the best models available for capturing the early stages of disease development, studying the effects of known risk factors, and testing new therapeutic approaches (Badiola-Mateos et al., 2021) (Figure 1 upper branch). However, as researchers create more complex and intricate models to replicate the structure and functionality of their *in-vivo* counterparts, our analytical tools must advance in sophistication to match. AI has proven to be fast, efficient, and quantitatively capable of extracting and analyzing the high-dimensional data generated from high-throughput organoid models, making it an ideal tool to deal with this type of data. Combining BOA with AI approaches (through an articulated synergy between AI variations and BOA architecture modeling) may thus lead to powerful hybrid systems (Shi et al., 2024). On the one hand, AI can be used to monitor the functioning of healthy (or diseased) BOAs, with the aim of testing new therapies or elucidating disease mechanisms. On the other hand, AI can also be exploited in hybrid AI/BOAs systems, in order to feed BOAs with appropriate stimuli and “train” them to respond, observing potential healing effects on brain diseases and aging. This scenario represents an unprecedented opportunity for both neuroscience and AI, offering a unique platform to approach cognitive processes objectively and systematically by using reproducible *in-vivo* human brain physiological systems, which can lead to entirely new types of preclinical studies and better tailored medical interventions. With this in mind, the fitting term *Organoid Intelligence* (OI), coined by Smirnova et al. (2023) by blending together AI and organoids, now describes a multidisciplinary field that aims at developing “biological computing using 3D cultures of human brain cells and brain-machine interface technologies.” This implies shifting from using machines to simulate brain activities [cf., von Neumann view (HP, 1959; Quirion, 2023) of “making computers brain-like”] to “growing the next supercomputer in a cell culture lab” (Quirion, 2023)—an opportunity that does not come without risks and poses novel methodological and ethical issues.

Albeit attractive, observing and measuring the functions of BOAs is not as straightforward as monitoring the activities of kidney or liver organoids. Functions that have an immediate–traceable–output, such as muscle contraction have been successfully replicated in organoids resembling cortex/spinal cord assemblies (Andersen et al., 2020), as are the effects of defective genes (Eichmüller and Knoblich, 2022). On the other hand, measuring BOAs responses linked to cognitive functions, such as learning and memory, is more challenging for both technical and ethical reasons. Yet, BOAs may represent a useful tool in which modeling and monitoring how specific neural assemblies learn and respond to chemicals or other factors that induce plastic changes, particularly because they grant for the possibility to be developed from the cells of a specific individual, leading to the creation of dynamic “personalized” models. Indeed, for living organisms, learning is a powerful instrument, and its impairment is one of the most dramatic consequences of disease. Through learning, systems can make and/or modify future responses based on past experiences: they can save energy by not responding to stimuli that have previously proven to be innocuous (habituation) and they can trigger responses in advance to stimulation by creating links between two or more stimuli that present in association (associative learning). Unsurprisingly, learning is a widespread phenomenon. Not only humans and animals can learn, but so can unicellular organisms (Tang and Marshall, 2018) and machines. By means of algorithms that associate different speech profiles to different labels, vocal assistants like Siri and Alexa learn to recognize the voice of the various speakers in a household and eventually address each member by his/her given name. Although we may view this behavior as if the assistant were “remembering” us (intentional instance; Dennett, 1988), the personalized approach results from a logic gate—not dissimilar from what happens in neural networks when cell discharge favors one of many possible pathways. In either case, learning develops as an implicit process, i.e., does not imply any form of conscious reflection on previous experiences or knowledge, but relies on adaptive modifications of behavior as a function of experience.

Recent studies suggest that this kind of learning can be observed in a 2D neuronal culture linked to a computer and that brain organoids may be trained to play a computer game (Kagan et al., 2022) or control movements of a robot (Schley, 2018). Although these findings do not indicate that cell cultures exhibit any form of sentient behavior (Friston, 2023), they provide a proof of concept that complex cognitive functions may be explored in brain organoids (BOAs), opening an entirely new realm of possibilities for research into neurological diseases, which can be described as “Assembloid learning.” In fact, one could envisage that in a near future, “personalized models” of how neural assemblies learn and undergo plastic changes could be developed, and used to assist brain care, for example by facilitating selection of the best treatment for each patient (e.g., by running *in vitro* trials first) (Figure 1). For this approach to be extended to functions such as memory, which lack a simple or unique measurable output, BOAs could benefit by hybridization with other approaches, like AI. Indeed, if proper AI approaches can be devised for optimizing the encoding/decoding of temporal-spatial information to and from BOAs (see Figure 1), it could be possible to elucidate how the individual brain responds to external factors and disease. In this scenario, an additional domain likely to benefit from this technique could be that of neurodevelopment, whose stages could be directly monitored, and the effects of potential threats explored at different times.

### Current limits hindering BOAs approaches

Though promising, the domain of BOA research is still in its infancy and several challenges remain to be addressed. Here, we will list the most prominent, indicating how current research may provide possible solutions.

First, developing BOAs and exploring how organoid intelligence works requires that these unique 3D cultures are correctly cultivated *in vitro* and their activities in response to input stimuli are successfully tracked. Neither pre-condition is easily satisfied. Despite the success of various protocols (Miura et al., 2020; Revah et al., 2022), organoids still suffer from low reproducibility, high heterogeneity, low generation throughput and necrosis/hypoxia. While heterogeneity may represent an advantage, allowing for the development of diverse neuronal connections and interactions (Sabate-Soler and Kurniawan, 2024) and promoting highly personalized treatment approaches, it is also a challenging aspect to control. Different culturing protocols, batch-to-batch variations, cell lines and/or gene expression, for example, can reduce coherence in the datasets, increasing the degree of unexplained heterogeneity, and hindering reproducibility and findings' reliability. Development of automated platforms (Jiang et al., 2020), adoption and sharing of standardized protocols, definition of the maturity state of the neural assemblies could certainly improve these limits.

Second, detecting BOAs activity is another critical issue. Fluorescence imaging methods, such as those using calcium indicators (Zhang et al., 2023) and voltage sensors (Evans et al., 2023), widely used to measure activity in neuronal populations, have limited temporal resolution and may suffer from photobleaching or phototoxicity. On the other hand, electrophysiological recordings can monitor neural activity over long intervals with high temporal resolution but may interfere with organoids cytoarchitecture and development due to direct contact with the neural substrate. Multielectrode arrays (MEAs) are a reasonable compromise, being versatile, and coming in different configurations (Sharf et al., 2022). A further promising opportunity comes from the innovative “Kirigami electronics” (KiriE) approach (Yang et al., 2023). Current platforms such as Brainoware (Cai et al., 2023) use flat and rigid MEA electrodes for interfacing with organoids but can only stimulate/record from a small number of neurons on the surface. In this respect, advantages could arise from further miniaturization of electrodes as well as re-arrangement of their topology. Alternatively, the way BOAs are grown could be changed, creating flat architectures that maintain the internal BOA network structure but can be accessed with standard MEA technology. Multimodal techniques for BOA interfacing should also be envisaged, exploring the possibility of integrating microfluidics, optical microscopy, optogenetics, and microfabrication to stimulate and read feedback from BOAs grown in a single platform.

Third, these stimulating opportunities, however, raise additional challenges. BOAs are living cells that require nutrition in more than one sense. Not only vascularization must be ensured but also power consumption of the infrastructure interfacing BOAs should be considered. Though intrinsically low, the addition of any complementary peripheral equipment (e.g., CO_2_ incubator) requires power, and technological solutions to decrease consumption should be developed, especially in virtue of a massive usage.

Fourth, novel and efficient modalities for data interpretation and visualization should be envisaged to deal with the large amount of information that can be extracted from these specimens. Graph theory-based methods (GTM) have recently proved reliable in partially understanding brain architecture, allowing for example to discriminate different forms of connectivity in fMRI studies (Farahani and Karwowski, 2019). However, cognitive, emotional, and behavioral responses are highly dynamic phenomena, both during development and in adult life. Presently, measures obtained via brain graph only display a snapshot of the disease over time, preventing immediate observation of the dynamics of brain networks (Fleischer et al., 2019). By extending the findings of fMRI studies (Madhyastha et al., 2018) and integrating GTM approach into the BOA environment, it would be possible to track the development of pathological conditions, as well as temporal correlations with topological alterations in the brain network.

Finally, but importantly, it is still a matter of debate how faithfully brain organoids replicate neural assemblies. A recent study for example (Bhaduri et al., 2020), reported that compared to primary cortical cells, single cells derived from organoids present differences in spatial organization and glial specificity—possibly due to ectopically activated cellular stress pathways. These observations suggest a word of caution, especially for studies on neurodevelopment, while, at the same time, indicate novel frameworks for improving the accuracy of cortical organoids (e.g., by acting on metabolic stress, transplantation), which may further benefit from the development of assembloids that may better capture the complexity of neural circuits (Miura et al., 2020, 2022).

## Discussion

Inevitably, the opportunity of developing brain models that can receive/process inputs and acquire implicit learning capabilities raises important ethical questions related to how these entities should be considered (Koplin and Savulescu, 2019; Lavazza, 2021; Jeziorski et al., 2023; Kagan et al., 2023a). Maturation of BOAs is accelerated by growth factors so that organoids at 10 weeks of culture show features (such as myelination) that in fetuses are viewed only after 20 weeks of gestation (Jakovcevski et al., 2009), instilling doubts as to whether they could potentially exhibit consciousness (Sawai et al., 2019) or experience rudimentary forms of pain. In fact, while developing brain organoids for modeling disease and/or testing novel drug appears both appealing and justified, the possible moral implications of novel technologies that may stem from this approach should also be proactively considered: for example, 2D-cell cultures have been already successfully connected to computers (Dennett, 1988) and organoids to muscle tissue (Andersen et al., 2020), opening to a plethora of enticing—but also ethically concerning possibilities. Recently, a debate ensued from mention in a paper that “*in vitro*, neurons learn and exhibit sentience” (Balci et al., 2023; Kagan et al., 2023b), suggesting that at least one major question should be promptly addressed. Although the nature and physiological mechanisms supporting consciousness is a puzzle that will not be rapidly solved, the forming OI community should consider exploring which definitions apply to culture models, qualifying and “translating” what is meant by “consciousness,” “sentience,” (Friston, 2023) or “awareness to stimuli” when BOAs are considered, and/or what terms should be used to describe more basic capacities, such as responding to sensory inputs (Friston, 2002; American Psychological Association, 2023). On this respect, a recent paper (Boyd, 2024) has proposed a series of qualities that should be considered when reasoning on the moral status of non-conventional entities—and that could represent a useful benchmark for dealing with neuroethical issues pertaining to brain organoids and assembloids.

Addressing issues related to the brain relies on a delicate equilibrium between enthusiasm for the potential benefits offered by biological computing in terms of improvements for public health, and a general fear of the unknown that can lead to skepticism and misinformation. Legal limitations, considerations based on ethics and moral, as well as evidence-based research, should provide the basis for establishing effective, ethical frameworks and principles to guide this vital work at the intersection of science, society, and policy, sidestepping the unfortunate challenges encountered with technologies like genetically modified organisms.

## Author contributions

AM: Writing—original draft, Writing—review & editing. ED: Writing—original draft, Writing—review & editing. DD-M: Writing—original draft, Writing—review & editing. FG: Writing—original draft, Writing—review & editing. FS: Writing—original draft, Writing—review & editing. EM: Writing—original draft, Writing—review & editing.
